# Multidimensional sleep impairment predicts steatotic liver disease spectrum risk

**DOI:** 10.1038/s41598-025-95336-9

**Published:** 2025-03-26

**Authors:** Dongling Wang, Xiao Zhang, Yujie Cai, Haihang Dong, Yinqiang Zhang

**Affiliations:** 1 Xiyuan Hospital, China Academy of Chinese Medical Sciences, Beijing, China; 2 Shanxi University of Chinese Medicine, Taiyuan, China

**Keywords:** Sleep pattern, Sleep duration, NAFLD, MAFLD, MASLD, Circadian rhythms and sleep, Neuroscience, Medical research

## Abstract

To evaluate the correlation between various sleep and the risk of NAFLD\MAFLD\MASLD. This study included 4772 subjects from the National Health and Nutrition Examination Survey data from 2017 to 2020. Poor sleep factors were defined as: ①abnormal sleep duration (< 7 h or > 8 h); ②snoring; ③sleep apnea; ④self-reported sleep disorder; ⑤ daytime sleepiness. The frequency of each sleep factor was scored, and the scores of all components were summed to obtain a sleep score ranging from 0 to 12. The higher the score, the less healthy the sleep pattern. Then we divided the overall sleep pattern into mild (sleep score 0–3 points), moderate (sleep score 4–7 points) or severe (sleep score 8–12 points) sleep pattern according to the distribution of sleep scores. Multiple logistic regression and restricted cubic spline graph analysis were used to determine the association between sleep and NAFLD\MAFLD\MASLD. In Model 1 and Model 2, sleep score as a continuous or categorical variable had an effect on NAFLD\MAFLD\MASLD(*p* <*0.05*). The risk of NAFLD\MAFLD\MASLD was higher in subjects with severe sleep patterns (*p* < *0.05*). Snoring and sleepy during day was associated with NAFLD\MAFLD\MASLD (*p* < *0.05*). We then drew a restricted cubic spline plot and found that sleep duration was nonlinearly associated with MAFLD\MASLD (*p* < *0.01*), and the risk of MAFLD\MASLD was lower when the sleep duration was 7.5 ~ 9.5 h/d. In this nationally representative survey, severe sleep patterns were associated with an increased risk of NAFLD/MAFLD/MASLD. It is worth noting that sleep duration was nonlinearly associated with MAFLD and MASLD.

**Supplementary Information:**

The online version contains supplementary material available at 10.1038/s41598-025-95336-9.

## Introduction

Non-alcoholic fatty liver disease (NAFLD) is a liver disease characterized by excessive fat deposition in hepatocytes in addition to prolonged heavy drinking and other definite liver injuries, and is one of the most common chronic liver diseases in the world, with a prevalence rate of about 25% among adults worldwide, and even up to 30% in the Western world^1,2^. With the deepening of understanding of fatty liver, researchers have gradually realized the limitations of the naming of NAFLD - the failure to clarify the correlation between metabolic disorders and the disease. In order to change the problem of alcohol stigmatization in patients with fatty liver, the Delphi consensus discussed by many experts was released in 2020, and NAFLD was renamed metabolic dysfunction-associated fatty liver disease (MAFLD)^3^. Since then, the diagnosis of fatty liver has changed from an exclusive diagnosis to an affirmative diagnostic criterion. In 2023, in order to further highlight metabolic cardiovascular risk factors and solve the stigma of “obesity” in patients with fatty liver, the name was changed to metabolic dysfunction-associated steatotic liver disease (MASLD), and drinking was further subdivided as an important pathogenic factor into Met-ALD and Pure-MASLD to clearly classify the spectrum of fatty liver disease^4^.

The quality of sleep is directly related to the normal functioning and recovery of the human body. Poor sleep quality is associated with a higher risk of all-cause and cause-specific mortality, the study noted^5^.Currently, sleep disorders have become a serious global challenge, accompanied by the rising incidence of breathing obstructions during the night. Studies have shown that there is a strong link between sleep and gut flora, which is one of the key pathways through which sleep regulates metabolism. Sleep disorders often lead to insulin resistance and abnormal lipid metabolism^6,7^, both of which are the pathological basis for the formation of fatty liver, and the frequent change in the name of fatty liver reflects researchers’ deep understanding of the role of metabolic factors in the disease. In addition, fatty liver is closely related to oxidative stress, and breathing obstructions during the night-induced respiratory impairment reduces oxygen intake and accelerates the deterioration of fatty liver to hepatitis^8^. Therefore, there is an inextricable link between sleep disorders and the development of fatty liver. Although studies have revealed the association between sleep disorders and metabolic diseases such as type 2 diabetes mellitus, hyperlipidemia^9^, hyperuricemia^10^etc., the specific mechanism of action between sleep and fatty liver has yet to be fully analyzed. In particular, comprehensive studies on the effects of factors such as sleep duration, snoring phenomenon and breathing obstructions during the night on fatty liver are still insufficient. In view of this, this research group comprehensively expounded the impact of sleep disorders on NAFLD/MAFLD/MASLD based on the cross-sectional data of NHANES 2017–2020. This study aims to investigate the association between sleep and fatty liver, address the potential limitations of previous studies, refine exposure factors such as sleep duration, snoring, sleep apnea syndrome, and study the three diseases together to provide data on the correlation between different sleep factors and fatty liver, and provide new insights for the management of patients with fatty liver disease.

## Materials & methods

### Study population

**Fig. 1:**
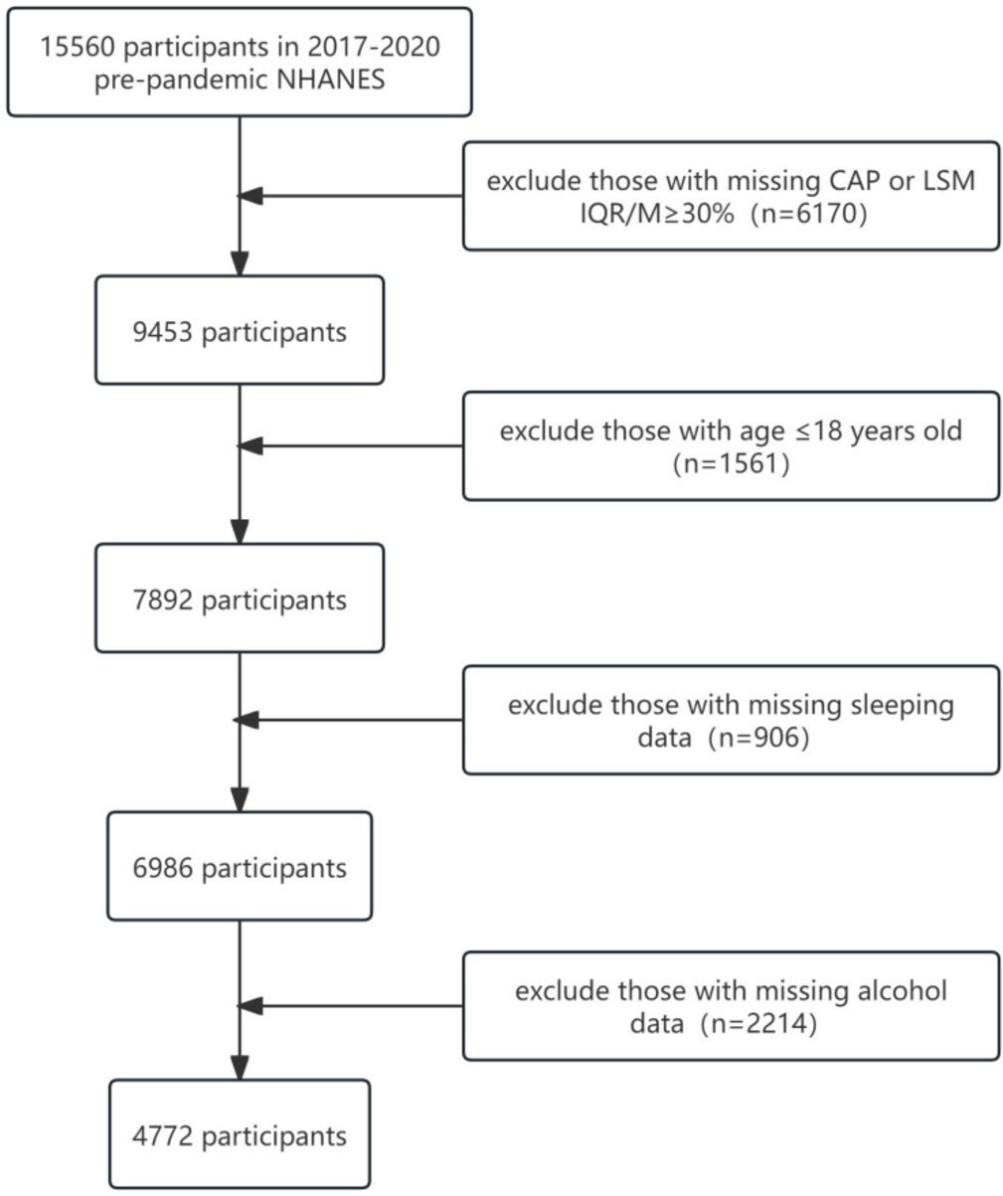
Research Sample Inclusion Process.

The National Health and Nutrition Examination Survey (NHANES) is a cross-sectional survey organized by the Centers for Disease Control and Prevention. Using a complex multi-stage probability sampling method and slecting a representative sample of the U.S. population to assess the health and nutritional status of the U.S. population. The Institutional Review Board of the National Center for Health Statistics approved this survey, and each participant signed an informed consent form before taking the questionnaire and laboratory tests. This study included data from the 2017–2020 NHANES, and all data were obtained from the NHANES website (https://www.cdc.gov/nchs/nhanes/index.htm). And our experimental procedure follows the STROBE guidelines^11^. Initially, a total of 15,560 samples were included. Participants who (1) lacked information on liver elasticity or poor test accuracy (*n* = 6170, 9453), (2) were aged ≤ 18 years (*n* = 1561, 7892), (3) lacked information on sleep (*n* = 906, 6986), and (4) lacked information on alcohol consumption (*n* = 2214, 4772) were excluded from further analysis. The final sample included in the analysis was 4772. The flow of the study samples is shown in Fig. 1.

## Measurement

### Outcome ascertainment

#### NAFLD

If the interquartile range/median of the participant’s LSM was < 30%, the examination accuracy was considered to be up to standard, and CAP ≥ 263 dB/m was used as the cutoff value for diagnosing steatosis liver diseases (SLD). NAFLD was defined as: (1) No excessive drinking (> 20 g/day for women and > 30 g/day for men. The drinking status of the participants was obtained through the drinking questionnaire. Excessive drinking was defined as > 2 cups/day for men and > 1 cup/day for women); (2) SLD participants without hepatitis B or hepatitis C infection (confirmed based on the participant’s medical history, drug treatment and corresponding laboratory tests).

## MAFLD

Based on SLD, combined with one of the following three criteria: overweight/obesity, type 2 diabetes and evidence of metabolic disorders. Metabolic disorders should include at least two of the following abnormalities: (1) waist circumference ≥ 102 cm for men and ≥ 88 cm for women; (2) blood pressure ≥ 130/85 mmHg or requiring corresponding drug treatment; (3) plasma triglycerides ≥ 1.70 mmoL/L (≥ 150 mg/dL), requiring special drug treatment; (4) plasma high-density lipoprotein cholesterol < 1.0 mmoL/L (< 40 mg/dL) for men and < 50 mg/dL (< 1.3 mmoL/L) for women, requiring special drug treatment; (5) fasting blood glucose level of 5.6 ~ 6.9 mmoL/L or glycated hemoglobin level of HbA1c of 5.7%~6.4%.; (6) insulin resistance score ≥ 2.5; (7) high-sensitivity C-reactive protein level > 2 mg/L.

### MASLD

The definition of MASLD is based on SLD and meets one of the following metabolic disorders: (1) BMI ≥ 25 kg/m2, or waist circumference ≥ 102 cm for men and ≥ 88 cm for women; (2) fasting blood glucose level of 5.6–6.9 mmoL/L or glycosylated hemoglobin (HbA1c) level of 5.7–6.4%, or a previous diagnosis of type 2 diabetes or current treatment for type 2 diabetes; (3) blood pressure level ≥ 130/85 mmHg or current treatment for hypertension; (4) individuals with triglyceride levels ≥ 1.70 mmol/L who require special drug treatment; (5) plasma high-density lipoprotein cholesterol < 1.0 mmoL/L (< 40 mg/dL) for men and < 50 mg/dL (< 1.3 mmoL/L) for women who require special drug treatment.

## Pure-MASLD and MetALD

Pure-MASLD was defined as MASLD without excessive alcohol consumption and other comorbid etiologies and MetALD with excessive alcohol consumption.

## Exposure measurement

Participants self-reported their sleep patterns on weekdays and weekdays. We included information from the sleep questionnaire on sleep duration on weekdays and weekends, snoring frequency, sleep apnea frequency, whether the patient had told their doctor about sleep difficulties and how often they felt excessively sleepy during the day. We calculated the sleep duration and obtained the usual sleep pattern:

$${\rm sleep \:duration} = ({\rm weekday\: sleep\: duration }\times 5 +{\rm weekend\: sleep\: duration\: \times 2)/7.}$$

The participants’ sleep duration was scored, with short or long sleep duration (< 7 h/d > 8 h) defined as 1 point. According to the joint consensus statement of the American Academy of Sleep Medicine and the Sleep Research Society on the recommended amount of sleep for healthy adults, the optimal healthy sleep duration for adults is 7 h^12^, so we classify short sleep as < 7 h/day, and the definition of long sleep varies, with most studies suggesting that sleep duration > 8 h/day is associated with increased all-cause mortality^12,13^. Therefore, after comprehensive consideration, we have defined < 7 h/d and > 8 h/d as unhealthy sleep duration. The sleep score consisted of five sleep factors (Sleep Pattern Scores = Sleep Duration + Trouble Sleeping + Snoring + Snort or Stop Breathing + Sleepy During Day) with a score range of 0 to 12 points, which was based on previous studies^12–14^, the specific calculation method is shown in Table 1. Higher scores indicated less healthy sleep patterns. We then classified the overall sleep pattern into healthy (sleep score 0–3 points), moderate (sleep score 4–7 points), or severe (sleep score 8–12 points) sleep patterns based on the distribution of sleep scores^15,16^.

**Table 1:** Sleep pattern scores composition.

Sleep pattern scores	Sleep duration	Trouble sleeping	Snoring	Snort or Stop breathing	Sleepy during day
0	7 ~ 8 h/d	No	Never	Never	Never
1	< 7 h/d or > 8 h/d	Yes	Rarely (1 ~ 2/week)	Rarely (1 ~ 2/week)	Rarely (1/month)
2	-	-	Occasionally (3 ~ 4/week)	Occasionally (3 ~ 4/week)	Sometimes (2 ~ 4/month)
3	-	-	Frequently (5 or more/week)	Frequently (5 or more/week)	Often (5 ~ 15/month)
4	-	-	-	-	Almost always (16 ~ 30/month)

### Covariates

Some potential confounding variables were taken into account, such as age, sex, cholesterol, triglycerides, education level was categorized as less than high school education, high school education and college education and above. Smoking status was categorized as former, current or never smoker. Sedentary time was selected as part of the physical activity questionnaire.

### Statistical analyses

NHANES provides weights for each participant to adjust the survey to represent the general U.S. population, taking into account design, nonresponse and post-stratification. The weighted population was used for analysis of sleep patterns, sleep duration and NAFLD\MAFLD\MASLD. Responses coded as “missing”, “refused” or “don’t know” in the original NHANES survey were considered missing. Participant characteristics were compared using chi-square tests or Wilcoxon rank-sum tests. For categorical variables, baseline information was presented as unweighted frequencies and weighted percentages; for continuous variables, weighted medians and quartiles were presented. To further explore the effects of covariates on sleep and NAFLD\MAFLD\MASLD, we used logistic regression to build Model 1 (crude adjustment: age, sex) and Model 2 (fully adjusted model: age, sex, race, education, smoking status and sedentary time). The weighted data were further used to draw restricted cubic spline plots with 4 nodes to analyze the association between different sleep durations and NAFLD\MAFLD\MASLD. R software (4.3.0) and stata (17.0) were used to collect and organize the data, and *P* < 0.05 was considered statistically significant for testing the research hypotheses.

## Results

### The baseline characteristics of study population

This research work included 4772 subjects (unweighted) at baseline assessment, and the overall prevalence of SLD, NAFLD, MAFLD, MASLD, and Pure-MASLD after weighted analysis was 47% (*n* = 71676232), 23% (*n* = 35,172,288), 45% (*n* = 68,547,574), 46% (*n* = 69,787,484) and 23% (*n* = 34,377,327) (Table 2). The risk of NAFLD, MAFLD and MASLD was higher in participants who were older, male, obese, had a higher waist circumference, had a longer sedentary time, and had a higher insulin resistance level, and the risk of MAFLD and MASLD was higher in participants with a higher total cholesterol level. The characteristics of the participants are summarized in more detail in Table S1.

**Table 2:** Prevalence of different types of fatty liver.

Group		*N*(%)
SLD		71,676,232 (47%)
NAFLD		35,172,288 (23%)
MAFLD		68,547,574 (45%)
MASLD		69,787,484 (46%)
Pure-MASLD		34,377,327 (23%)
A	NAFLD+/MAFLD+/MASLD+/Pure-MASLD+	33,651,704 (22%)
B	NAFLD+/MAFLD-/MASLD+/Pure-MASLD+	725,624 (0.5%)
C	NAFLD-/MAFLD+/MASLD+/Pure-MASLD-	34,874,809 (23%)
D	NAFLD+/MAFLD-/MASLD-/Pure-MASLD-	794,961 (0.5%)
E	NAFLD-/MAFLD-/MASLD-/Pure-MASLD-	81,181,986 (53%)

SLD, steatotic liver disease; MAFLD, dysfunction-associated fatty liver disease; MASLD, metabolic dysfunction-associated steatotic liver disease; NAFLD, non-alcoholic fatty liver disease metabolic.

### The association between sleep pattern and risk of NAFLD\MAFLD\MASLD

**Fig. 2:**
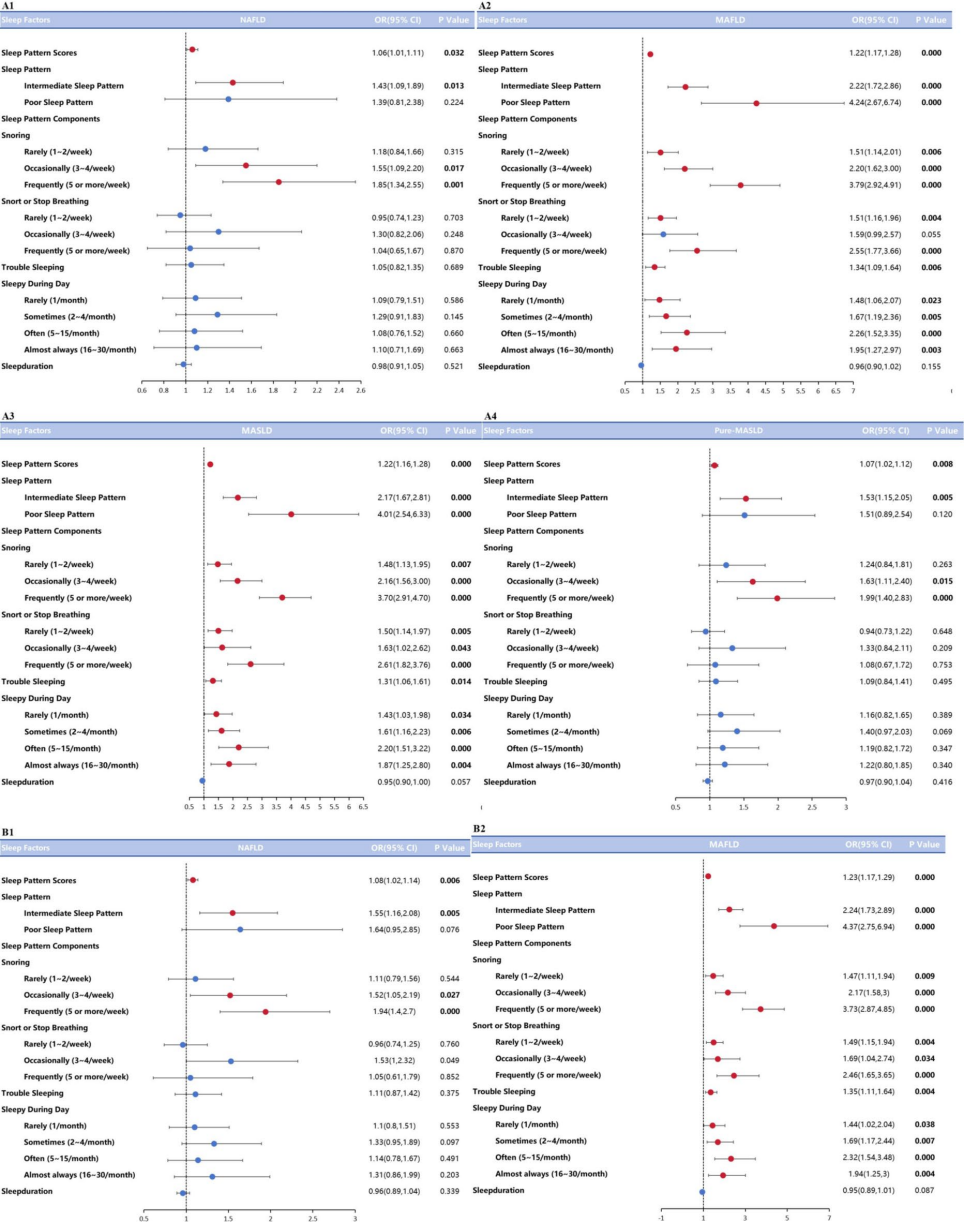

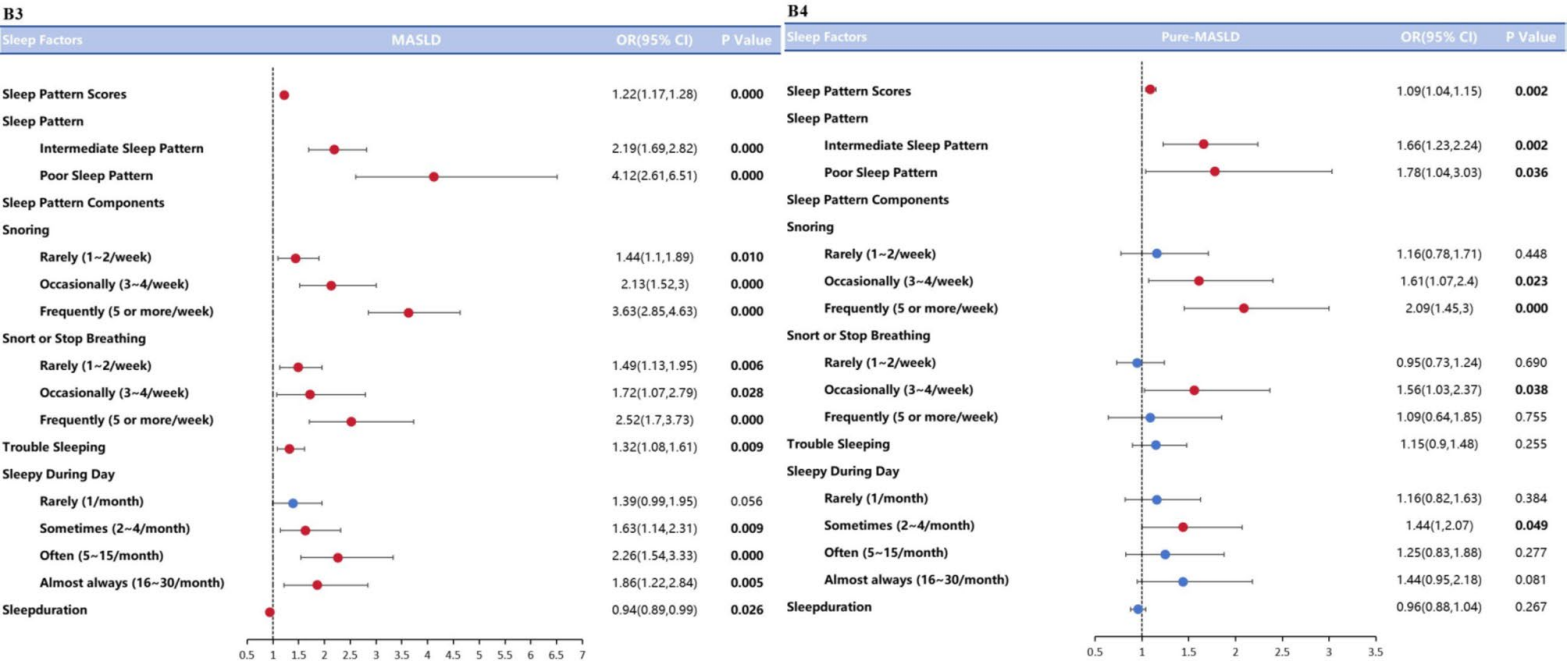
The association between sleep pattern and NAFLD\MAFLD\MASLD\Pure-MASLD. A1 ~ A4are Model1 (we adjust age and gender). A1: The association between sleep pattern and NAFLD; A2: The association between sleep pattern and MAFLD; A3: The association between sleep pattern and MASLD; A4: The association between sleep pattern and Pure-MASLD. B1 ~ B4 are Model 2 (we adjust age, gender, race, education, smoking status, sedentary time). B1: The association between sleep pattern and NAFLD; B2: The association between sleep pattern and MAFLD; B3: The association between sleep pattern and MASLD; B4: The association between sleep pattern and Pure-MASLD. Blue box means OR value without statistical difference. Red box means OR value with statistical difference. The bars on both sides of box mean 95% CI of OR. CI, confidence intervals; OR, odds ratio.

As shown in Figs. 2A1 to A4, in the age- and gender-adjusted model (Model 1), sleep score as either a continuous or categorical variable had an effect on NAFLD\MAFLD\MASLD\Pure-MASLD\MetALD (*p* < *0.05*). The subjects with moderate sleep patterns were 1.43, 2.22, 2.17, and 1.53 times more likely to develop NAFLD\MAFLD\MASLD\Pure-MASLD compared to subjects with mild patterns (*p* < *0.05*). Subjects with severe sleep patterns were 4.24, 4.01 times more likely to have MAFLD\MASLD than subjects with healthier sleep patterns (*p* < *0.001*). After adjusting for potential confounders (Model 2), as in Fig. 2B1 to B4 sleep scores still had an effect on NAFLD\MAFLD\MASLD\Pure-MASLD (*p* < *0.05*). Moderate sleep patterns had an effect on NAFLD (1.55(1.16,2.08)), MAFLD (2.24(1.73,2.89)),MASLD (2.19(1.69,2.82)),Pure-MASLD (1.66(1.23,2.24)) (*p* < *0.01*), and severe sleep pattern subjects with MAFLD\ MASLD\Pure-MASLD were 4.37, 4.12, and 1.78 times higher than those of mild sleep pattern individuals (*p* < *0.001*).

We then investigated the correlation between the components of sleep patterns and NAFLD\MAFLD\MASLD\Pure-MASLD, and the results showed that snoring associated with NAFLD\MAFLD\MASLD\Pure-MASLD in both Model 1 and Model 2 (*p* < *0.05*), and that as the frequency of snoring increased, the risk of NAFLD\MAFLD\Pure-MASLD increased. MASLD\Pure-MASLD the risk of developing NAFLD\MAFLD\Pure-MASLD also gradually increased. Reported sleep disturbances were associated with MAFLD\MASLD (*p* < *0.05*, *p* < 0.01), and apnea during sleep and daytime sleepiness were associated with MAFLD\MASLD (*p* < *0.05*, *p* < 0.01), which remained statistically significant after fully adjusting for modeling (*p* < *0.05*, *p* < 0.01). In model 1, sleep duration was not associated with NAFLD\MAFLD\MASLD\Pure-MASLD, and in model 2, it was associated only with MASLD (*p* < *0.05*). The specific model comparison data are shown in Table 3.

**Table 3:** The association between sleep pattern and NAFLD\MAFLD\MASLD\Pure-MASLD.

Sleep Factors	OR(95% CI)								
	NAFLD		MAFLD		MASLD		Pure-MASLD		
	Model 1	Model 2	Model 1	Model 2	Model 1	Model 2	Model 1	Model 2	
**Sleep Pattern Scores**	1.06(1.01,1.11)	1.08(1.02,1.14)	1.22(1.17,1.28)	1.23(1.17,1.29)	1.22(1.16,1.28)	1.22(1.17,1.28)	1.07(1.02,1.12)	1.09(1.04,1.15)	
**Sleep Pattern**									
**Intermediate Sleep Pattern**	1.43(1.09,1.89)	1.55(1.16,2.08)	2.22(1.72,2.86)	2.24(1.73,2.89)	2.17(1.67,2.81)	2.19(1.69,2.82)	1.53(1.15,2.05)	1.66(1.23,2.24)	
**Poor Sleep Pattern**	1.39(0.81,2.38)	1.64(0.95,2.85)	4.24(2.67,6.74)	4.37(2.75,6.94)	4.01(2.54,6.33)	4.12(2.61,6.51)	1.51(0.89,2.54)	1.78(1.04,3.03)	
**Sleep Pattern Components**									
**Snoring**									
**Rarely (1 ~ 2/week)**	1.18(0.84,1.66)	1.11(0.79,1.56)	1.51(1.14,2.01)	1.47(1.11,1.94)	1.48(1.13,1.95)	1.44(1.1,1.89)	1.24(0.84,1.81)	1.16(0.78,1.71)	
**Occasionally (3 ~ 4/week)**	1.55(1.09,2.20)	1.52(1.05,2.19)	2.20(1.62,3.00)	2.17(1.58,3)	2.16(1.56,3.00)	2.13(1.52,3)	1.63(1.11,2.40)	1.61(1.07,2.4)	
**Frequently (5 or more/week)**	1.85(1.34,2.55)	1.94(1.4,2.7)	3.79(2.92,4.91)	3.73(2.87,4.85)	3.70(2.91,4.70)	3.63(2.85,4.63)	1.99(1.40,2.83)	2.09(1.45,3)	
**Snort or Stop Breathing**									
**Rarely (1 ~ 2/week)**	0.95(0.74,1.23)	0.96(0.74,1.25)	1.51(1.16,1.96)	1.49(1.15,1.94)	1.50(1.14,1.97)	1.49(1.13,1.95)	0.94(0.73,1.22)	0.95(0.73,1.24)	
**Occasionally (3 ~ 4/week)**	1.30(0.82,2.06)	1.53(1,2.32)	1.59(0.99,2.57)	1.69(1.04,2.74)	1.63(1.02,2.62)	1.72(1.07,2.79)	1.33(0.84,2.11)	1.56(1.03,2.37)	
**Frequently (5 or more/week)**	1.04(0.65,1.67)	1.05(0.61,1.79)	2.55(1.77,3.66)	2.46(1.65,3.65)	2.61(1.82,3.76)	2.52(1.7,3.73)	1.08(0.67,1.72)	1.09(0.64,1.85)	
**Trouble Sleeping**	1.05(0.82,1.35)	1.11(0.87,1.42)	1.34(1.09,1.64)	1.35(1.11,1.64)	1.31(1.06,1.61)	1.32(1.08,1.61)	1.09(0.84,1.41)	1.15(0.9,1.48)	
**Sleepy During Day**									
**Rarely (1/month)**	1.09(0.79,1.51)	1.1(0.8,1.51)	1.48(1.06,2.07)	1.44(1.02,2.04)	1.43(1.03,1.98)	1.39(0.99,1.95)	1.16(0.82,1.65)	1.16(0.82,1.63)	
**Sometimes (2 ~ 4/month)**	1.29(0.91,1.83)	1.33(0.95,1.89)	1.67(1.19,2.36)	1.69(1.17,2.44)	1.61(1.16,2.23)	1.63(1.14,2.31)	1.40(0.97,2.03)	1.44(1,2.07)	
**Often (5 ~ 15/month)**	1.08(0.76,1.52)	1.14(0.78,1.67)	2.26(1.52,3.35)	2.32(1.54,3.48)	2.20(1.51,3.22)	2.26(1.54,3.33)	1.19(0.82,1.72)	1.25(0.83,1.88)	
**Almost always (16 ~ 30/month)**	1.10(0.71,1.69)	1.31(0.86,1.99)	1.95(1.27,2.97)	1.94(1.25,3)	1.87(1.25,2.80)	1.86(1.22,2.84)	1.22(0.80,1.85)	1.44(0.95,2.18)	
**Sleepduration**	0.98(0.91,1.05)	0.96(0.89,1.04)	0.96(0.90,1.02)	0.95(0.89,1.01)	0.95(0.90,1.00)	0.94(0.89,0.99)	0.97(0.90,1.04)	0.96(0.88,1.04)	

### The association between sleep duration and risk of NAFLD\MAFLD\MASLD

**Fig. 3:**
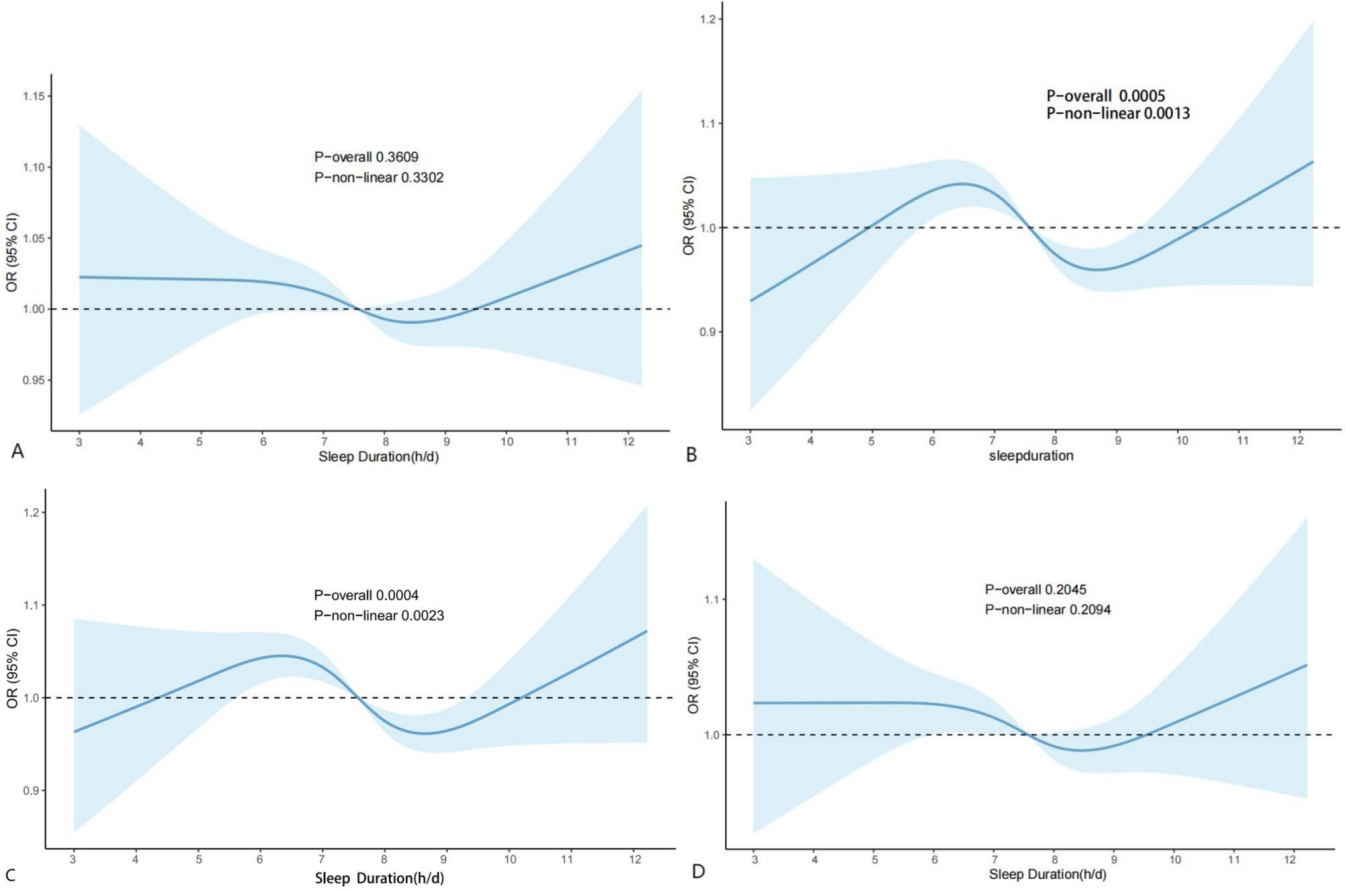
The association between sleep duration and risk of NAFLD\MAFLD\MASLD\Pure-MASLD. **A**: The association between sleep duration and risk of NAFLD; **B**: The association between sleep duration and risk of MAFLD; **C**: The association between sleep duration and risk of MASLD; **D**: The association between sleep duration and risk of Pure-MASLD. We adjust age, gender, race, education, smoking status, sedentary time.

In order to study the correlation between sleep duration and NAFLD\MAFLD\MASLD\Pure-MASLD, a restricted cubic spline diagram was subsequently drawn (Fig. 3). As shown in Fig. 3B ~ C, sleep duration was nonlinearly associated with MAFLD\MASLD (*p* < *0.01*), and the risk of MAFLD\MASLD was lower when the sleep duration was 7.5 ~ 9.5 h/d.

**Fig. 4:**
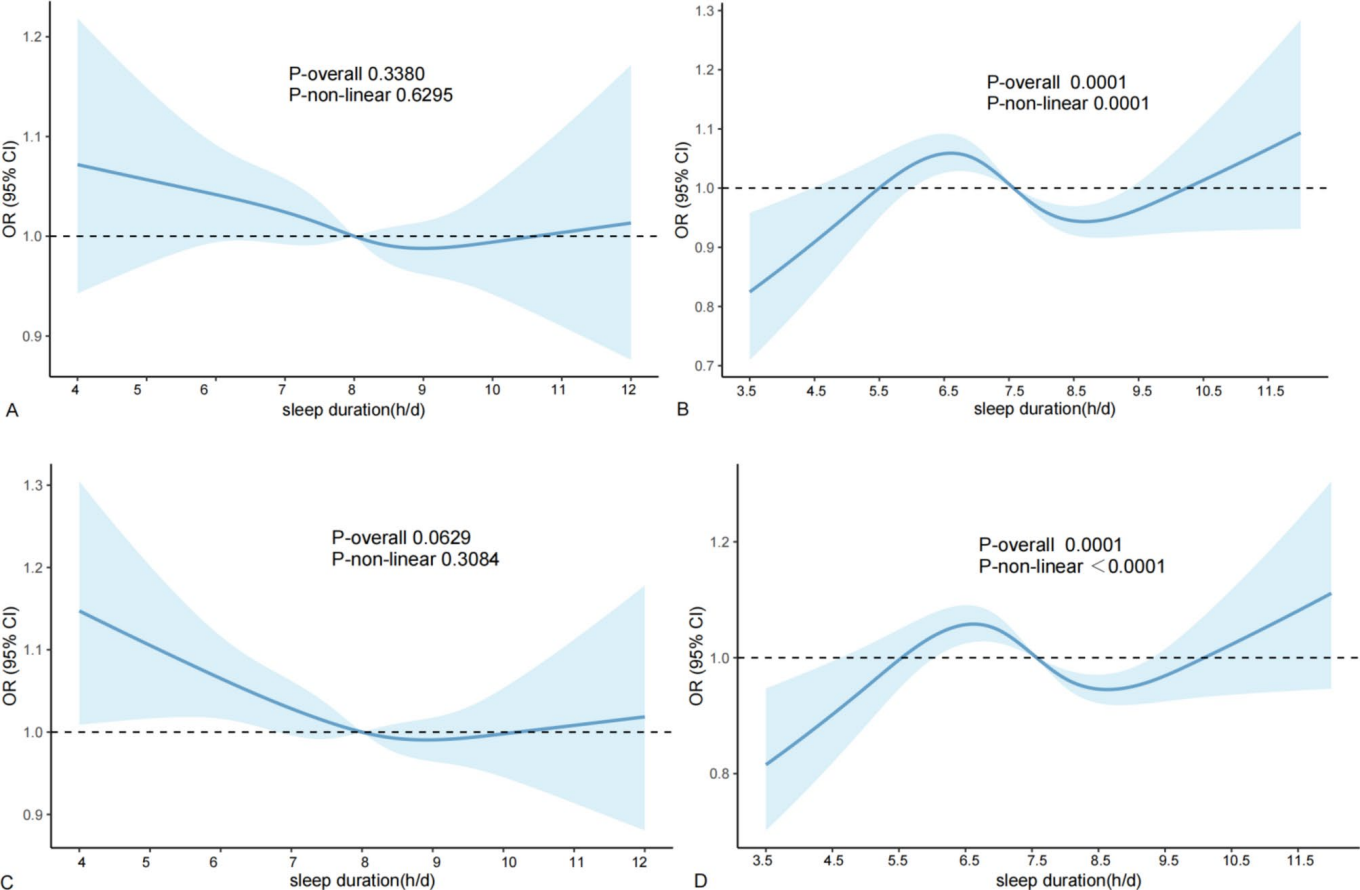
The association between sleep duration and MAFLD/MASLD in different gender groups. **A**: The association between sleep duration and MAFLD in women; **B**: The association between sleep duration and MAFLD in men; **C**: The association between sleep duration and MASLD in women; **D**: The association between sleep duration and Pure-MASLD in men. We adjust age, race, education, smoking status, sedentary time.

Subsequently, we investigated the relationship between sleep and MAFLD/MASLD by gender. The results showed that the nonlinear correlation between sleep duration and MAFLD/MASLD in men remained significant (*p* < 0.001), but sleep duration had no significant effect on women (*p*>0.05), as shown in Fig. 4.

## Discussion

As an important factor affecting metabolism, sleep and its relationship with fatty liver deserve further exploration. On this basis, we first studied the data of fatty liver under several different diagnostic criteria and the related issues of specific sleep problems. Based on the US Health and Nutrition Database, we evaluated the clinical characteristics of fatty liver under these different definitions and their relationship with sleep patterns. The main findings of our analysis are: (1) The total prevalence of NAFLD, MAFLD, MASLD and Pure-MASLD was 23%, 45%, 46% and 23% respectively (Table 2). Due to the broader definition of metabolic risk factors, the prevalence of MASLD and MAFLD was higher than that of NAFLD, which to a certain extent promoted the active diagnosis and treatment of a part of fatty liver patients that were previously ignored; (2) The definition of pure-MASLD is similar to that of NAFLD, so the results of the two are relatively close. Both emphasize the exclusive feature of “non-alcoholic”, but this feature may bring confusion and limitations in actual diagnosis. However, this relatively exclusive diagnosis can help us unravel the mystery, accurately discover the specific pathogenic factors, and help patients with fatty liver whose metabolic function has not yet declined to find the cause. (3) The effects of sleep on MAFLD and MASLD are similar, so there is a nonlinear correlation between sleep duration and both, and the results of the two are similar. This similarity may stem from the shared emphasis of MAFLD and MASLD on metabolic dysfunction as a core diagnostic criterion. Both definitions prioritize the presence of metabolic risk factors (e.g., obesity, insulin resistance, dyslipidemia), which are closely linked to sleep disturbances. In contrast, NAFLD’s diagnostic framework is based on the exclusion of alcohol consumption and other secondary causes, without explicitly incorporating metabolic dysfunction. This distinction may explain why sleep duration shows a more pronounced association with MASLD and MAFLD, as their pathogenesis is more directly influenced by metabolic alterations driven by poor sleep. Additionally, MASLD specifically emphasizes the presence of significant hepatic fibrosis, which may amplify the measurable impact of sleep disturbances. Chronic sleep deprivation or poor sleep quality exacerbates systemic inflammation and oxidative stress, which are key drivers of fibrogenesis in MASLD. This could explain why sleep duration demonstrates a clearer association with MASLD compared to NAFLD or MAFLD, where fibrosis may not yet be prominent. The shared metabolic focus of MAFLD and MASLD also likely contributes to their similar results, as both conditions are more sensitive to sleep-related metabolic dysregulation than the broader NAFLD definition. (4) There is a nonlinear correlation between sleep duration and MAFLD/MASLD in men, whereas in women, this correlation is not present. This may be related to the estrogen secreted by females, which has anti-inflammatory effects and improves insulin sensitivity, potentially buffering the metabolic negative effects of long sleep. In males, the lack of testosterone can lead to visceral fat accumulation, insulin resistance, and dyslipidemia, and sleep deprivation can reduce testosterone secretion, forming a vicious cycle of metabolic disorder^17^. Estrogen lowers plasma cholesterol and low-density lipoprotein, increases high-density lipoprotein, inhibits atherosclerosis, hepatic lipid deposition, and the release of inflammatory factors from adipose tissue, and alleviates hepatic oxidative stress^18^.

Xiaoyu Wang^19^ found that more frequent insomnia symptoms were significantly associated with higher triglyceride-glucose index. This study found that the higher the sleep score, the worse the sleep, the higher the risk of NAFLD\MAFLD\MASLD\Pure-MASLD (*p* < *0.05*). The moderate sleep pattern promotes the occurrence of NAFLD\MAFLD\MASLD\Pure-MASLD (*p* < *0.01*), subjects with severe sleep patterns suffer from MAFLD\MASLD\Pure-MASLD which are 4.37, 4.12, and 3.23 times more likely than individuals with mild sleep patterns (*p* < *0.001*). This shows that the worse the sleep pattern of patients, the higher the risk of fatty liver. Reduced sleep quality leads to the activation of NF-κB and MCP-1^20^, promotes the production of inflammatory cytokines (such as IL-6, IL-8, TNF-α)^21^, recruits monocytes and other inflammatory cells to local liver tissues, and these inflammatory factors further aggravate the inflammatory response of the liver, and also affect the insulin signaling pathway, leading to the occurrence of insulin resistance, aggravating the deposition and degeneration of fat in the liver, and leading to the occurrence and aggravation of fatty liver.

We then analyzed the correlation between each sleep factor and fatty liver and found that in Model 1 and Model 2, snoring was associated with NAFLD\MAFLD\MASLD\Pure-MASLD. As the frequency of snoring increased, the risk of NAFLD\MAFLD\MASLD\Pure-MASLD gradually increased, and sleep apnea was associated with MAFLD\MASLD. Mouth breathing by subjects with breathing obstructions during the night will affect the oral humidity and flora, leading to disturbances in the oral and intestinal flora, and abnormal metabolism of the oral-liver-intestinal axis^22^. HiAlcKpn bacteria caused by breathing obstructions during the night will lead to excessive endogenous alcohol production and aggravate the degree of FLD^23^. Other studies^24–26^ have pointed out that intermittent nocturnal hypoxemia caused by reported breathing obstructions during the night is an important mechanism for increased sympathetic nerve activity and upregulation of systemic inflammation, and intermittent hypoxia also induces inflammation in adipose tissue, causing weight gain and metabolic disorders, leading to obesity, and further aggravation of IR. This study found that sleep disorders are associated with MAFLD\MASLD (*p* < *0.05*, *p* < 0.01). Sleep disorders can increase sympathetic nerve excitability, which in turn causes vasoconstriction and increased blood pressure, affecting the blood supply to the liver, leading to liver ischemia, hypoxia and other problems. Insufficient sleep can promote the release of stress hormones such as cortisol, inhibit the liver’s detoxification function, lead to the accumulation of harmful substances in the body, and increase liver metabolic pressure. To study the nonlinear relationship between sleep duration and fatty liver, we drew a restricted cubic bar graph. The results showed that sleep duration was nonlinearly associated with MAFLD\MASLD (*p* < *0.01*). The risk of MAFLD\MASLD was lower when the sleep duration was between 7.5 and 9 h/d. The sleep duration is U-shaped related to MAFLD/MASLD, so we speculate that both excessively long and short sleep durations can lead to the development of MAFLD/MASLD, although the statistical results in this article showed *P*> 0.05. Previous studies have shown that reduced sleep time is associated with the prevalence of fatty liver. Oxidative stress is one of the key factors in the pathogenesis and progression of fatty liver. It promotes the occurrence and development of fatty liver by affecting mitochondrial function^27^, lipid peroxidation, protein damage, DNA damage, and inflammatory response. Insufficient sleep duration will lead to increased accumulation of oxygen free radicals (ROS) in the body, exacerbating oxidative stress response, and oxidative stress interferes with normal sleep by increasing neuronal activity and causing cell damage. Observational studies have shown that some inflammatory markers such as IL-6 and TNF-α are elevated in long sleepers^28^, and the production of inflammatory cytokines (such as IL-6, TNF-α) will recruit monocytes and other inflammatory cells to local liver tissues, leading to further aggravation of the liver’s inflammatory response^29^, and also affecting the insulin signaling pathway, leading to the occurrence of insulin resistance^30^, aggravating the deposition and degeneration of fat in the liver, and leading to the occurrence and aggravation of fatty liver. The continuous release of inflammatory factors in a long-term insomnia state can promote the activation of hepatic stellate cells and secrete collagen, leading to liver tissue fibrosis. If not treated in time, it may develop into cirrhosis, at which time the liver structure is permanently changed and loses its normal function.

Compared with previous studies, we analyzed fatty liver and sleep quality under various definitions for the first time based on the US Nutrition and Health Database, explored the correlation between sleep and fatty liver, and concluded that sleep disorders are risk factors for fatty liver, which emphasizes the important potential role of good sleep in preventing the risk of fatty liver. Clinically, our findings suggest that sleep assessment should be incorporated into routine screening protocols for individuals at risk of fatty liver disease. Healthcare providers should consider asking patients about sleep duration, snoring frequency, and breathing obstructions during clinical evaluations. For patients diagnosed with both fatty liver and sleep disorders, multidisciplinary management involving hepatologists and sleep medicine specialists could be implemented to design personalized interventions, such as cognitive behavioral therapy for insomnia or continuous positive airway pressure (CPAP) therapy, which may synergistically improve metabolic outcomes.

In addition, our study also has some limitations: First, the collection of sleep data is based on self-reports of subjects, and there may be recall bias in the data. Obstructive sleep apnea is defined as more than 5 apnea, hypoxia, or sleep-wake events per hour despite trying to breathe^31^. Most patients with OSA were diagnosed by clinical prediction tools, self-questionnaires, and polysomnography, and the OSA patients collected in this paper were also self-reported. The diagnosis of OSA itself is somewhat subjective, so the diagnosis of OSA patients collected in the NHANES database is a reference. In subsequent data collection, polysomnography validation can be selected to make the data as objective as possible. Furthermore, this study is a cross-sectional study, which cannot establish a causal relationship between sleep and fatty liver. Future research should design longitudinal studies, conduct follow-up studies on the study population, or design controlled studies to validate the findings of this study. A prospective cohort design will be implemented, establishing a baseline cohort based on high-risk populations for sleep disorders identified by current research (such as patients with OSA and individuals with insomnia). This cohort will be followed for ≥ 5 years, with at least annual follow-ups. Stratification will be conducted according to fatty liver subtypes (NAFLD/MAFLD/MASLD, etc.) and sleep disorder types (OSA, insomnia, circadian rhythm disorders) to ensure statistical power for subgroup analyses. In addition to conventional metabolic indicators (ALT, AST, hepatic steatosis index), objective data from polysomnography (PSG) (AHI, blood oxygen saturation), and inflammatory markers (IL-6, TNF-α) will be integrated. Fecal metagenomics (gut microbiota), plasma metabolomics, and single-cell transcriptomics (markers of hepatic stellate cell activation) will be collected before and after interventions to reveal the molecular mechanisms underlying sleep interventions. Additionally, the incomplete racial analysis is also a significant limitation. Since we selected the US National Health and Nutrition Examination Survey (NHANES) database, the included population does not represent Asia or Europe, and therefore the analysis results cannot be generalized to the entire population. In the future, integrating databases from multiple countries can be considered to make the research results more representative.

## Conclusion

Severe sleep patterns, especially snoring, were significantly associated with increased prevalence of NAFLD\MAFLD\MASLD\Pure-MASLD, and sleep duration was nonlinearly associated with MAFLD\MASLD.

## Electronic supplementary material

Below is the link to the electronic supplementary material.

Supplementary Material 1

## Data Availability

The data used in this study were available in the NHANES public database: https://www.cdc.gov/nchs/nhanes/index.htm.
