# Supplementary material for: Multidimensional sleep impairment predicts steatotic liver disease spectrum risk
Source: Sci Rep. 2025 Mar 26;15:10405. doi: 10.1038/s41598-025-95336-9 (PMC11947187; doi:10.1038/s41598-025-95336-9)
Supplement: Supplementary file 1 — Supplementary Material 1 [file 41598_2025_95336_MOESM1_ESM.docx]

**Table S1 The baseline characteristics of study population.**

| **Characteristic** | **Overall** | | **Nafld** | | | **Mafld** | | | **Masld** | | | **MetALD** | | | **Pure-MASLD** | | |
| --- | --- | --- | --- | --- | --- | --- | --- | --- | --- | --- | --- | --- | --- | --- | --- | --- | --- |
|  | N1 | Overall, N = 4772 (100%)2 | **No,N = 3611 (77%)2** | **Yes,N = 1161 (23%)2** | p-value3 | **No,N = 2552 (55%)2** | **Yes,N = 2220 (45%)2** | p-value3 | **No,N = 2507 (54%)2** | **Yes,N = 2265 (46%)2** | p-value3 | **No,N = 3665 (77%)2** | **Yes,N = 1107 (23%)2** | p-value3 | **No,N = 3642 (77%)2** | **Yes,N = 1130 (23%)2** | p-value3 |
| **Age** | 4,772 | 44 (31, 58) | 41 (29, 56) | 53 (39, 65) | <0.001 | 40 (28, 56) | 48 (35, 60) | <0.001 | 39 (28, 56) | 49 (36, 60) | <0.001 | 43 (30, 59) | 45 (33, 56) | 0.9 | 41 (29, 56) | 53 (39, 64) | <0.001 |
| **Sex** | 4,772 |  |  |  | <0.001 |  |  | <0.001 |  |  | <0.001 |  |  | 0.4 |  |  | <0.001 |
| Male |  | 2,501 (51%) | 1,763 (47%) | 738 (65%) |  | 1,221 (45%) | 1,280 (58%) |  | 1,195 (45%) | 1,306 (58%) |  | 1,933 (51%) | 568 (50%) |  | 1,782 (47%) | 719 (65%) |  |
| Female |  | 2,271 (49%) | 1,848 (53%) | 423 (35%) |  | 1,331 (55%) | 940 (42%) |  | 1,312 (55%) | 959 (42%) |  | 1,732 (49%) | 539 (50%) |  | 1,860 (53%) | 411 (35%) |  |
| **Race** | 4,772 |  |  |  | 0.028 |  |  | <0.001 |  |  | <0.001 |  |  | <0.001 |  |  | 0.071 |
| Mexican American |  | 601 (8.8%) | 451 (8.7%) | 150 (9.1%) |  | 218 (6.2%) | 383 (12%) |  | 214 (6.2%) | 387 (12%) |  | 364 (7.1%) | 237 (15%) |  | 453 (8.7%) | 148 (9.2%) |  |
| Other Hispanic |  | 489 (7.2%) | 382 (7.6%) | 107 (5.7%) |  | 247 (7.2%) | 242 (7.2%) |  | 246 (7.3%) | 243 (7.1%) |  | 351 (6.8%) | 138 (8.6%) |  | 385 (7.6%) | 104 (5.7%) |  |
| Non-Hispanic White |  | 1,720 (65%) | 1,269 (65%) | 451 (67%) |  | 908 (67%) | 812 (64%) |  | 889 (66%) | 831 (64%) |  | 1,342 (67%) | 378 (60%) |  | 1,280 (65%) | 440 (67%) |  |
| Non-Hispanic Black |  | 1,238 (10%) | 984 (11%) | 254 (8.8%) |  | 755 (12%) | 483 (8.8%) |  | 748 (12%) | 490 (8.7%) |  | 1,004 (11%) | 234 (8.7%) |  | 989 (11%) | 249 (8.8%) |  |
| Non-Hispanic Asian |  | 470 (4.4%) | 327 (4.0%) | 143 (5.7%) |  | 287 (4.9%) | 183 (3.7%) |  | 274 (4.8%) | 196 (3.9%) |  | 413 (5.0%) | 57 (2.4%) |  | 336 (4.1%) | 134 (5.4%) |  |
| Other Race-Including Multi-Racial |  | 254 (3.9%) | 198 (4.1%) | 56 (3.4%) |  | 137 (3.4%) | 117 (4.6%) |  | 136 (3.4%) | 118 (4.5%) |  | 191 (3.4%) | 63 (5.7%) |  | 199 (4.1%) | 55 (3.5%) |  |
| **Education** | 4,655 |  |  |  | 0.3 |  |  | <0.001 |  |  | <0.001 |  |  | <0.001 |  |  | 0.4 |
| Less than 9th grade |  | 219 (2.2%) | 158 (2.2%) | 61 (2.2%) |  | 80 (1.7%) | 139 (2.8%) |  | 80 (1.7%) | 139 (2.8%) |  | 141 (1.8%) | 78 (3.4%) |  | 159 (2.2%) | 60 (2.2%) |  |
| 9-11th grade |  | 432 (5.7%) | 342 (6.1%) | 90 (4.7%) |  | 224 (5.2%) | 208 (6.4%) |  | 216 (5.1%) | 216 (6.5%) |  | 302 (4.9%) | 130 (8.5%) |  | 346 (6.1%) | 86 (4.6%) |  |
| High school graduate/GED or equivalent |  | 1,081 (25%) | 847 (26%) | 234 (23%) |  | 555 (24%) | 526 (28%) |  | 545 (24%) | 536 (28%) |  | 787 (24%) | 294 (31%) |  | 850 (26%) | 231 (24%) |  |
| Some college or AA degree |  | 1,633 (31%) | 1,233 (31%) | 400 (32%) |  | 855 (30%) | 778 (33%) |  | 843 (30%) | 790 (33%) |  | 1,243 (31%) | 390 (33%) |  | 1,245 (31%) | 388 (32%) |  |
| College graduate or above |  | 1,290 (35%) | 925 (35%) | 365 (38%) |  | 745 (39%) | 545 (31%) |  | 730 (39%) | 560 (31%) |  | 1,089 (39%) | 201 (24%) |  | 935 (35%) | 355 (38%) |  |
| **Smoking status** | 4,770 |  |  |  | <0.001 |  |  | <0.001 |  |  | <0.001 |  |  | <0.001 |  |  | <0.001 |
| Never smoker |  | 2,697 (57%) | 1,996 (55%) | 701 (63%) |  | 1,492 (58%) | 1,205 (55%) |  | 1,470 (58%) | 1,227 (55%) |  | 2,159 (60%) | 538 (47%) |  | 2,016 (55%) | 681 (63%) |  |
| Former smoker |  | 1,124 (26%) | 777 (25%) | 347 (30%) |  | 507 (23%) | 617 (30%) |  | 494 (23%) | 630 (30%) |  | 842 (25%) | 282 (29%) |  | 786 (25%) | 338 (30%) |  |
| Current smoker |  | 949 (17%) | 836 (20%) | 113 (6.8%) |  | 552 (19%) | 397 (15%) |  | 542 (19%) | 407 (15%) |  | 663 (15%) | 286 (24%) |  | 838 (20%) | 111 (6.8%) |  |
| **BMI** | 4,742 | 28 (24, 33) | 27 (24, 32) | 32 (28, 36) | <0.001 | 25 (23, 29) | 32 (29, 37) | <0.001 | 25 (23, 29) | 32 (29, 37) | <0.001 | 27 (24, 31) | 33 (29, 37) | <0.001 | 27 (24, 32) | 32 (28, 36) | <0.001 |
| **Waist** | 4,684 | 98 (88, 110) | 95 (85, 107) | 108 (99, 118) | <0.001 | 90 (81, 98) | 109 (100, 119) | <0.001 | 90 (81, 98) | 109 (100, 119) | <0.001 | 95 (85, 106) | 109 (99, 119) | <0.001 | 95 (85, 106) | 108 (100, 118) | <0.001 |
| **Sedentary duration** | 4,764 | 300 (180, 480) | 300 (180, 480) | 360 (240, 480) | 0.007 | 300 (180, 480) | 360 (240, 480) | <0.001 | 300 (180, 480) | 360 (240, 480) | 0.002 | 300 (180, 480) | 300 (180, 480) | 0.7 | 300 (180, 480) | 360 (240, 480) | 0.005 |
| **Sleep pattern** | 4,772 |  |  |  | 0.001 |  |  | <0.001 |  |  | <0.001 |  |  | <0.001 |  |  | <0.001 |
| healthy |  | 1,639 (34%) | 1,313 (37%) | 326 (26%) |  | 1,092 (43%) | 547 (24%) |  | 1,069 (43%) | 570 (24%) |  | 1,379 (38%) | 260 (23%) |  | 1,333 (37%) | 306 (25%) |  |
| intermediate |  | 2,830 (60%) | 2,087 (58%) | 743 (67%) |  | 1,368 (53%) | 1,462 (68%) |  | 1,346 (53%) | 1,484 (68%) |  | 2,100 (58%) | 730 (68%) |  | 2,098 (58%) | 732 (68%) |  |
| poor |  | 303 (5.4%) | 211 (5.1%) | 92 (6.5%) |  | 92 (3.2%) | 211 (8.2%) |  | 92 (3.2%) | 211 (8.0%) |  | 186 (4.3%) | 117 (9.1%) |  | 211 (5.1%) | 92 (6.6%) |  |
| **Triglyceride** | 4,488 | 1.24 (0.87, 1.85) | 1.14 (0.81, 1.73) | 1.61 (1.14, 2.33) | <0.001 | 1.02 (0.76, 1.41) | 1.64 (1.16, 2.33) | <0.001 | 1.02 (0.76, 1.41) | 1.64 (1.14, 2.33) | <0.001 | 1.14 (0.84, 1.72) | 1.64 (1.13, 2.28) | <0.001 | 1.14 (0.81, 1.73) | 1.63 (1.16, 2.33) | <0.001 |
| **Total cholesterol** | 4,501 | 4.73 (4.11, 5.51) | 4.73 (4.11, 5.48) | 4.81 (4.14, 5.56) | 0.6 | 4.65 (4.06, 5.38) | 4.89 (4.22, 5.66) | 0.002 | 4.65 (4.06, 5.38) | 4.89 (4.22, 5.66) | 0.001 | 4.68 (4.09, 5.43) | 4.99 (4.29, 5.72) | <0.001 | 4.73 (4.11, 5.48) | 4.81 (4.14, 5.56) | 0.5 |
| **HOMA-IR** | 2,221 | 2.10 (1.31, 3.85) | 1.82 (1.19, 3.09) | 3.38 (2.10, 5.48) | <0.001 | 1.50 (1.01, 2.40) | 3.38 (2.10, 5.78) | <0.001 | 1.50 (1.01, 2.41) | 3.35 (2.09, 5.71) | <0.001 | 1.88 (1.20, 3.29) | 3.10 (1.87, 5.80) | <0.001 | 1.80 (1.19, 3.09) | 3.54 (2.24, 5.70) | <0.001 |
| 1 N not Missing (unweighted) | | | | | | | | | | | | | | | | | |
| 2 Median (IQR); n (unweighted) (%) | | | | | | | | | | | | | | | | | |
| 3 Wilcoxon rank-sum test for complex survey samples; chi-squared test with Rao & Scott’s second-order correction | | | | | | | | | | | | | | | | | |
